# Supplementary material for: Analysis of Tissue-Specific Defense Responses to Sclerotinia sclerotiorum in Brassica napus
Source: Plants (Basel). 2022 Jul 31;11(15):2001. doi: 10.3390/plants11152001 (PMC9370628; doi:10.3390/plants11152001)
Supplement: Supplementary file 1 [file plants-11-02001-s001.zip › plants-1826220-supplementary/plants-1826220_supplemnetary-done.pdf]

plants MDPI

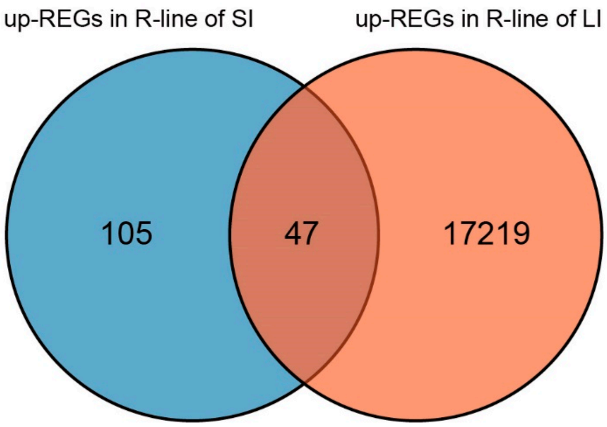

Figure S1. Unique and common up-REG in R-line of SI and LI.

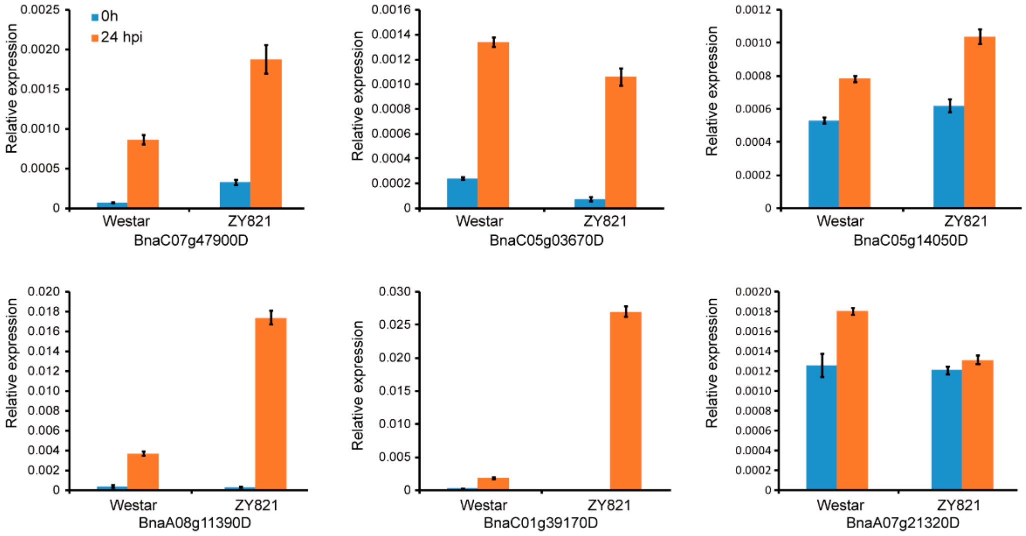

Figure S2. Transcriptome data validation by qRT-PCR of six genes.
